# Mathematical Models as Tools to Predict the Release Kinetic of Fluorescein from Lyotropic Colloidal Liquid Crystals

**DOI:** 10.3390/ma12050693

**Published:** 2019-02-26

**Authors:** Donatella Paolino, Andra Tudose, Christian Celia, Luisa Di Marzio, Felisa Cilurzo, Constantin Mircioiu

**Affiliations:** 1 Department of Experimental and Clinical Medicine, University of Catanzaro “Magna Graecia”, Viale “S. Venuta” s.n.c., 88100 Catanzaro, Italy; paolino@unicz.it (D.P.); andratds@yahoo.com (A.T.); 2 Department of Applied Mathematics and Biostatistics, Faculty of Pharmacy, University of Medicine and Pharmacy “Carol Davila” Bucharest, 6 Traian Vuia, 020956 Bucharest, Romania; 3 Department of Pharmacy, University of Chieti—Pescara “G. d’Annunzio”, via dei Vestini 31, 66100 Chieti, Italy; luisa.dimarzio@unich.it (L.D.M.); felisa.cilurzo@unich.it (F.C.)

**Keywords:** lyotropic colloidal liquid crystals, release profile, mathematical models, drug delivery systems, release kinetic, square root laws

## Abstract

In this study, we investigated the release kinetic of fluorescein from colloidal liquid crystals made from monoglyceride and different non-ionic surfactants. The crystals were physicochemically characterized and the release experiments were carried out under the sink conditions, while mathematical models were described as extrapolations from solutions of the diffusion equation, in different initial and boundary conditions imposed by pharmaceutical formulations. The diffusion equation was solved using Laplace and Fourier transformed functions for release kinetics from infinite reservoirs in a semi-infinite medium. Solutions represents a general square root law and can be applied for the release kinetic of fluorescein from lyotropic colloidal liquid crystals. Akaike, Schwartz, and Imbimbo criteria were used to establish the appropriate mathematical model and the hierarchy of the performances of different models applied to the release experiments. The Fisher statistic test was applied to obtain the significance of differences among mathematical models. Differences of mathematical criteria demonstrated that small or no significant statistic differences were carried out between the various applied models and colloidal formulations. Phenomenological models were preferred over the empirical and semi-empirical ones. The general square root model shows that the diffusion-controlled release of fluorescein is the mathematical models extrapolated for lyotropic colloidal liquid crystals.

## 1. Introduction

The release kinetic of drugs from pharmaceutical formulations plays a main role in the study of biopharmaceutical features of payloads following their administration into the body [1]. In fact, the process of drug release from pharmaceutical formulations can modulate drug absorption into the various biological compartments; besides its distribution and plasma concentration, that is the rate of absorption and bioavailability in biological fluids [2]. In this attempt, technological approaches are extensively used to design pharmaceutical formulations and to obtain a controlled release of drugs. Furthermore, drug release is affected by different physiological parameters, e.g., pH of the gastric tract, bile and pancreatic secretion, and drug dissolution from formulations [3]. Other parameters, such as physicochemical properties of biocompatible materials, their chemical structures, the thickness of polymeric or lipid shells, the interaction between drugs and plasmatic proteins, the coefficient of permeation and diffusion might also affect the release profile of drug delivery systems [4,5].

The release profile of drugs and bio-molecules from conventional and innovative carriers, such as lyotropic colloidal liquid crystals, could be described using mathematical approaches. In fact, liquid crystals can be arranged in different supramolecular structures, due to the self-assembling of surfactants [6]. Their geometrical arrangement, as well as their interaction with biological substrates, depends on the thermodynamic transition from solid to liquid state of biomaterials that modulates potential applications in nanomedicine and biotechnology [7]. Colloidal liquid crystals can exist as thermotropic and lyotropic mesophases, and their transition from first to second supramolecular structure depends on the ionic interactions between polar head groups of surfactants, hydrophobic interactions between hydrocarbon tails, surfactant concentration in water, and critical micellar concentration. All these parameters can modify the arrangement of surfactants and lipid components in lyotropic colloidal liquid crystals, thus, modifying their physicochemical and biopharmaceutical properties [8,9].

In this attempt, preliminary in vitro experiments are needed to evaluate the release profile of payloads from lyotropic colloidal liquid crystals and to predict their potential in vivo behavior. The prediction of physicochemical properties in colloidal formulations can be investigated using mathematical models that suitably describe the potential in vitro/in vivo trend [10,11].

In the last decade, several scientists showed that different mathematical models, in particular the Higuchi’s law, can be applied, to theoretically calculate the release of drugs from colloidal nanoparticles made from organic, inorganic or hybrid materials [12]. This model was used in specific experimental conditions where the release of payloads depends on the diffusion of solvent through a homogeneous matrix, and cannot be used to predict mathematical models describing the release of drugs from colloidal nanoparticles-based surfactants, like lyotropic liquid crystals. This evidence should be true for surfactants representing the main components of pharmaceutical formulations [6]. The excess of surfactants makes a reservoir, which entraps drugs and slowly releases the payloads, during this time. This effect allows a release kinetic, depending on the square root of the time, which is similar to the kinetic profile of nanoparticles [13]. The resulting kinetic model is similar to Higuchi’s law; in fact, the use of pure Higuchi’s law to study the kinetic release profile of drugs might generate a mismatch model, during the analysis.

Different formulations have been designed by combining glyceryl mono-oleate derivatives, or polyethylene glycol hexadecyl ether/polyoxyethylene cetyl ether, or copolymers composed of a central hydrophobic chain of polyoxypropylene (poly(propylene oxide)), flanked by two hydrophilic chains of polyoxyethylene (poly(ethylene oxide)) (symperonic and poloxamers, or pluronic, of different molecular weight), to evaluate the impact of lyotropic colloidal liquid crystal composition and physicochemical properties on the theoretical and experimental release kinetic profile of payloads. Polyethylene glycol oleyl ether (Brij^®^ 92) and polyoxyethylene stearyl ether (Brij^®^ 72) are not used as controls but like copolymers similar to polyethylene glycol conjugated to fatty acids, and are combined with various polyethylene polypropylene glycol derivatives (pluronic and symperonic). In fact, Brij 92 is polyethylene glycol oleyl ether, polyoxyethylene (2) oleyl ether (or Brij O2), while Brij^®^ 72 is polyoxyethylene (2) stearyl ether. These two surfactants showed the presence of saturated and unsaturated fatty acids, respectively, in their backbone structure, and Brij 92 can sometimes be used as a control for other formulations, containg mono-oleate derivatives, with respect to Brij 72, because it has in its backbone structure the oleyl derivate, which is a saturated fatty acid similar to oleate of monomuls, instead of stearyl unsaturated fatty acid.

In this work, the massive release kinetics of drugs, resulting from the experimental data, has been described using different mathematical models, such as Noyes–Whitney [14], square root model [15], Siepman–Peppas [16], and Weibull [17]. The comparison of different mathematical methods has been carried out by using the information criteria. The mathematical models reported, herein, supported the resulting data and demonstrated that the phenomenological models were preferred over the empirical and semi-empirical ones, for estimation of the release kinetic of hydrophilic drugs from lyotropic colloidal liquid crystals.

The square root model has been referred to as the Higuchi model, in this paper, but finally, it has been underlined that there are two different sets of phenomenological conditions that lead to such a law.

## 2. Materials and Methods

### 2.1. Materials

Fluorescein hydrochloride (fluorescein) was purchased from Sigma-Aldrich Italy (Milan, Italy). Polyoxyethylene-polyoxypropylene block copolymer 127 (Symperonic^®^ PE/F127) was obtained from Uniquema, Croda Italiana S.p.A. (Mortara (PV) Italy). Polyethylene glycol-polypropylene glycol-polyethylene glycol block copolymer 6800 (Pluronic^®^ PE 6800), polyethylene glycol block copolymer 10500 (Pluronic^®^ PE 10500), Polyoxyethylene stearyl ether (Brij^®^ 72), Polyethylene glycol oleyl ether (Brij^®^ 92) were received as a gift from ACEF Spa (Piacenza, Italy). Glyceryl mono-oleate (Monomuls^®^ 90-O18) was obtained from Cognis S.p.A. (Fino Mornasco (CO), Italy). Double-distilled pyrogen-free water was purchased from Sifra S.p.A. (Verona, Italy). Isotonic sterile saline solution (NaCl 0.9% w/v) was a product of Fresenius Kabi Potenza S.r.l. (Verona, Italy). All other materials and solvents were of analytical grade and were used without any further purification (Carlo Erba, Milan, Italy).

### 2.2. Lyotropic Colloidal Liquid Crystals

Lyotropic colloidal liquid crystals were synthesized by emulsifying hydrophobic surfactants with an aqueous solution of a poly(ethylene) glycol derivative. The mixing-dilution procedure was carried out to obtain different formulations. Monomuls (90 mg) was dissolved in ethanol (2 mL) and the hydrophilic surfactants were dissolved in distilled water or isotonic saline solution (NaCl 0.9% w/v) (8 mL). The aqueous phase was added drop-by-drop to the organic phase, under continuous stirring, using an Ultraturrax T25 basic homogenizer (IKA^®^-Werke GmbH & Co. KG, Staufen, Germany), at a mixing speed of 18,500 rpm (3 different cycles of 5 min). The formulation was formed at room temperature. An inclusion complex was obtained when the co-surfactant was added to the ternary system ethanol-mono-olein-water, thus, affecting the supramolecular structure of lyotropic colloidal liquid crystals. The formulations were stored at room temperature, for 48 h, under continuous stirring (400 rpm), in order to remove any residual trace of ethanol. The residual and not assembling surfactants were then removed by using a dialysis membrane with a molecular cut-off of 50,000 Dalton (Spectra/Por Membranes, Spectrum Laboratories, Inc., Rancho Dominguez, CA, USA). The dialysis was performed for 48 h, at room temperature, using sterile isotonic saline solution (NaCl 0.9% w/v) (200 mL) as receptor medium. A constant mixing rate of 400 rpm was maintained during purification, and saline solution were replaced every 8 h. The dialysis of fluorescein-loaded lyotropic colloidal liquid crystals was carried out in a dark atmosphere to preserve the fluorescin entrapped in the various formulations from potential light degradation. Four different formulations were prepared as reported in Table 1. Aqueous fluorescein solution (0.03% w/v), used as a hydrophilic drug model, during experiments, was added to the aqueous phase during the preparation procedure; in particular, fluorescein was dissolved in the distilled water and added to Monomuls and surfactants, drop-by-drop, under continuous stirring of the ethanol solution of the lipid components, by using an Ultraturrax T25 basic homogenizer (IKA^®^-Werke GmbH & Co. KG, Staufen, Germany), at a mixing speed of 18,500 rpm (3 different cycles of 5 min). The vial tube used to make fluorescein lyotropic colloidal liquid crystals was sealed using aluminum foils and the preparation of nanoformulations was carried out in a dark atmosphere to avoid the light degradation of fluorescein.

### 2.3. Physicochemical Characterization of Lyotropic Colloidal Liquid Crystals

The physicochemical characterization of lyotropic colloidal liquid crystals was carried out using a Zetasizer Nano ZS (Malvern Instruments Ltd., Worchestershire, UK). Photon correlation spectroscopy was applied to measure the average sizes and size distribution of lyotropic colloidal liquid crystals, while Doppler laser anemometry and, hence, the electrophoretic mobility to measure the Z-potential values, as previously reported [18,19]. Zetasizer Nano ZS was set-up, according to the following instrumental parameters, for the measurement of average sizes and narrow size distribution: laser diode of 4.5 mW, operating at 670 nm as a light, back scattering angle at 173°; real refractive index 1.59, imaginary refractive index 0.0, medium refractive index 1.330, medium viscosity 1.0 mPa·s, medium dielectric constant of 80.4 (Malvern Instruments Ltd., Worchestershire, UK). A Smoluchowsky constant F (Ka) of 1.5; He/Ne laser doppler anemometry (633 nm), with a nominal power of 5.0 mW, were used to measure the Z-potential (Malvern Instruments Ltd., Worchestershire, UK). Samples were diluted 1:100 (v/v) with isotonic buffer, pre-filtered through polypropylene membranes with a pore size of 0.22 μm (Whatman Inc., Clifton, NJ, USA), before the analysis, to avoid the multiscattering phenomenon. Lyotropic colloidal liquid crystals were held into disposable polystyrene cells, for sizes and size distribution, and disposable folded capillary cells for Z-potential (Malvern Instruments Ltd., Worchestershire, UK), to measure their physicochemical parameters.

The entrapment efficiency of lyotropic colloidal liquid crystals was carried out, using the ultracentrifugation method. Samples were centrifuged at 100,000× *g*, 4 °C, 1 h, with a Beckman Optima^™^ ultracentrifuge equipped with a TL S55 fixed-angle rotor (Beckman Coulter Inc., Fullerton, CA, USA). Lyotropic colloidal liquid crystals were then analyzed, using a UV-Vis spectrophotometer (Perkin Elmer Lamda 25, Norwalk, CT, USA), at the maximum wavelength of 480 nm. The entrapment efficiency percentage of various samples was measured, according to the following equation:

EE (%) = [(D_T_ − D_U_)/D_T_] × 100, (1)

where D_T_ represents the total amount of fluorescein added to lyotropic colloidal liquid crystals, during the preparation procedure, while D_U_ is the amount of payload in the supernatant.

The amount of fluorescein initially added to different lyotropic colloidal liquid crystals was 0.03% (w/v) and corresponded to 3.75 mg/mL.

### 2.4. Release Experiments

The release of fluorescein from lyotropic colloidal liquid crystals was investigated using a dialysis membrane. Regenerated cellulose membrane with a molecular cut-off of 25,000 Dalton (Spectra/Por Membranes, Spectrum Laboratories, Inc., Rancho Dominguez, CA, USA) was used for the release experiments. Membranes were hydrated, before analysis, for 40 min, using a sterile isotonic saline solution to remove the traces of sodium azide storage solvent. Membranes were then filled with 1 mL of different formulations, sealed with dialysis clips, and then placed in a pyrex glass beaker containing 200 mL of sterile isotonic saline solution. Experiments were carried out at room temperature for 24 h and sink conditions were maintained during the analysis. At different incubation times, 1 mL of the receptor medium was withdrawn, replaced with the same volume of fresh isotonic saline solution and then immediately analyzed using an UV-Vis spectrophotometer (Perkin Elmer Lambda 20, Norwalk, CT, USA). A fluorescein calibration curve was used to quantify the payload released from the lyotropic liquid crystals, according to the following equation:

*y* = 0.88 *x* + 0.148, (2)

where, *x* represents the fluorescein concentration (µg/ml) and *y* the UV/Vis absorbance (nm). The *r*^2^ value was 0.9982. No interference was observed at fluorescein λ_max_ of 495 nm from other components of formulation.

### 2.5. Statistical Criteria and Information on Selection of Mathematical Models

#### 2.5.1. Akaike and Schwarz criteria

The Akaike information criterion (AIC) [20] and Schwarz criterion (SC) [21] were the two different mathematical methods applied to analyze data. Both of these models are based on the addition of statistical errors corrected by a penalty function, which are proportional to the number of parameters (*p*) evaluated in the following models:

(3) AIC=N ln SS+2p,

(4) SC=N ln SS+p lnN,

where, *N* represents the number of point data, and squared errors *SS* is the weighted sum of squared deviations of a model with a set of *p* parameters, calculated according to the following equation:

(5) $$SS=\sum_{i=1}^{n}w_{i}{\left(y_{i}^{exp}-y_{i}^{calc}\right)}^{2},$$

where, *W_i_* is the weighting factor for the respective data.

The model equation having the lowest AIC or SC were selected for the evaluation of the time course plots [22].

#### 2.5.2. Imbimbo Criterion

The Imbimbo criterion is based on the mean area between the limits of a 90% confidence interval for calculated values, according to the model, $\hat{y_{i}}=y_{i}^{calc}$ [23] and using the following equation:

(6) $$Ip=\frac{t_{\left(N-p,0.05\right)}}{\bar{y_{i}^{calc}}}\sqrt{SS_{p}\left(\frac{1}{\left(N-p\right)}-\frac{1}{N}\right)},$$

where, $\bar{y_{i}^{calc}}$ is the mean of estimated concentrations versus time, $t_{\left(N-p,0.05\right)}$ is 0.05 quintile for Student distribution with *N*−*p* degree of freedom, and *SS_p_* is the above-mentioned *SS* in the case of models with *p* parameters.

In fact the index is approximately the ratio between area of the confidence limits and area under a theoretical curve. The model equation with the minimum *I_p_* value generates the narrowest confidence interval for the estimated amounts of released drug from different formulations.

#### 2.5.3. Fisher (F) Test Criterion

We can compare a simple model having *q* parameters with a complex model having supplementary *k* parameters, with *p* = *q* + *k* using the *F* ratio, according to the following equation:

(7) $$F=\frac{SS_{q}-SS_{p}}{SS_{p}}\;\frac{df_{p}}{df_{q}-df_{p}},$$

where, *SS_q_* is the sum of standard errors for the selected reference mathematical model; while *SS_p_* corresponds to the more complex model. The number of freedom degrees represents the difference between the number of experimental data, *n*, and the number of parameters:

(8) $$df_{p}=n-p\;\mathrm{and}\;df_{q}=n-q,$$

The analysis makes significance when the two models are nested, i.e., the model with a lower number of parameters can be considered as degenerated from the model with more parameters, by keeping the number of parameters constant a.

### 2.6. Applied Mathematical Models

#### 2.6.1. Zero Order Model

Zero order release kinetics described a constant release of payloads and this mathematical model reported a constant release kinetic of drug in plasma, as well as biological fluids. The following general equation was applied for a constant release kinetic:

*R*(*t*) = *a* + *kt*, (9)

where, *R* is the percent release of payloads.

#### 2.6.2. Noyes–Whitney Model

A linear dependence can be obtained by transforming the previous equation by a logarithmic transformation, as reported below:

(10) −ln(1−r(t))=kt,

where, *r*(*t*) is the fraction released at time *t*, calculated as a percentage of the quantity of payloads that are released during the time.

The delivery of payloads was calculated across a limit stationary layer of thickness (*δ*), which appeared in the receptor solution at the border with lyotropic liquid crystals, which was not affected by the convection currents. The concentration gradient is usually considered linear in this limit layer. Furthermore, the concentration of payloads was equal to its maximum value *Cs*. Parameters reported herein represent the concentration of payloads at the stationary layer (*Cδ*) and its solubility at the maximum concentration in the medium (*S*). The constant *k* was proportional with the diffusion coefficient at the interface and area *A*, as reported in the following equation:

(11) $$k=\frac{AD}{\delta },$$

The initial and boundary conditions for *C*(*x*,*t*) were *C*(0,*t*) = *Cs*, and *C(δ*,*t*) = *Cδ* (*t*) for *x* ≥ *δ*.

#### 2.6.3. Weibull Model

Equation (10) can be further transformed into a linear model, by applying the double logarithmic transformation, as reported in the following equation:

(12) ln(−ln(1−R/100))=lnα+βln(t),

where, *α* and *β* are empirical constants.

Langerbucher first applied this model for describing the dissolution of drugs from pharmaceutical formulations, by using the Weibull probability distribution function [17]; recently, it has been applied to analyze the dissolution and release of drugs from pharmaceutical formulations in different experimental conditions [20,21,22,23,24].

In different simulations [24] of power laws, the Weibull function and the fitting of experimental data of diltiazem and diclofenac [10] demonstrated that the exponent β, for polymeric matrices, is an indicator of the mechanism of transport for the drug through the polymer matrix. A value of β ≤ 0.75 was associated with the Fick diffusion in either fractal or Euclidian spaces, while a combined mechanism (Fick diffusion and swelling controlled transport) was associated with β values in the range 0.75 < β < 1.

For β values over 1, it was demonstrated that the drug transport shows a complex release mechanism.

#### 2.6.4. Power Law Equation (Siepman–Peppas) Model

The release kinetic profile of payloads from pharmaceutical formulations in a specific drug range of concentration was analyzed using a power law equation proposed by Siepman and Peppas [16]. This mathematical model included, both, the effects of diffusion and the erosion of drug from colloidal systems as reported in the following Equation:

(13) $$r(t)=\alpha t^{\beta },$$

The following mathematical model represents a generalization of Higuchi’s law, and it could be considered a degeneration of the Weibull model, for low values of time.

#### 2.6.5. Construction of Diffusion Models by using Fick’s Second Law

Fick’s second law predicts how the diffusion process can modify the concentration of drugs over time. The dependence between the drug concentration and the time is described by the following Equation:

(14) $$\frac{\partial c}{\partial t}=D\frac{\partial^{2}c}{\partial x^{2}},$$

where, *c* represents the concentration of payloads at point *x*, *t* is time, *D* is the diffusion coefficient.

An infinite number of solutions can be obtained by using Fick’s second law, as reported in Supplementary information section (Equations S2–S10).

#### 2.6.6. Higuchi Square Root Law

Higuchi applied Fick’s first law to describe the release of drugs in a limit layer, at the surface of a pharmaceutical matrix (e.g., ointment, tablet) toward an external solvent, which acts as a perfect sink under pseudo steady-state conditions. Since the assumptions of the model are approved only in the first part of the release process, the application of this law is recommended only for the first 60% of the release curve [25]. By evaluating the release profiles from ointments and insoluble matrices, Higuchi’s law is expressed as a square root function, as reported below:

(15) $$m=\sqrt{DC_{s}\left(2C_{0}-C_{s}\right)t}=\alpha t^{1/2},$$

where, *D* is the diffusion coefficient, *C*_0_ is the initial drug concentration in the matrix, and *C_S_* the solubility of the drug.

#### 2.6.7. Square Root Laws

A similar square root law to the Higuchi equation was further used to describe the release kinetic of drugs from pharmaceutical formulations, which can be considered as an infinite reservoir at the interface with a large or semi-infinite solution [26,27,28]. The concentration of drugs inside solution can be expressed, using the following equation:

(16) $$c(y,t)=c_{s}(1-erf\left(\frac{y}{\sqrt{4Dt}}\right)),$$

where, *y* is the distance from the interface and *erf* (*z*) is the error function calculated as the area under the curve $\frac{2}{\sqrt{\pi }}$$e^{-x^{2}}$, with the limit between 0 and *z*:

(17) $$erf(z)=\int_{0}^{z}\frac{2}{\sqrt{\pi }}e^{-x^{2}}dx,$$

The integration of flux of the interface in the range between 0 and *t* further provided the following square root equation:

(18) $$m(t)=\frac{2}{\sqrt{\pi }}Ac_{s}\sqrt{Dt},$$

where, *A* is the area of interface between the reservoir and the diffusion medium.

The square root of time laws could arise in a more general frame, as reported in the Supplementary information section (Equations S2–S10).

### 2.7. Graphical Representation of Data

SPSS v.14.0 Software (GraphPad Software, Inc., San Diego, CA, USA) was used for the graphical representation of results. These data represent the average of three different experiments ± standard deviation.

## 3. Results and Discussion

Drug delivery systems have different release kinetic profiles, which depended on the physicochemical properties of drugs. Polymeric suspensions and the colloidal carriers provided a release kinetic and profiles similar to various pharmaceutical formulations, and a zero or first order kinetic was obtained during the experiments. The application of mathematical criteria in the controlled release of drugs from supramolecular-, micro-, and nano- carriers depended on some mechanisms, and the model selected. Three phenomena could explain the mechanism of release of drugs from lyotropic colloidal liquid crystals, and particularly: (i) the diffusion-controlled, (ii) the swelling-controlled, and (iii) the chemical-controlled release [29].

Different mathematical models have been extensively proposed to analyze the release and dissolution kinetics of drugs from pharmaceutical formulations [24,30].

The release kinetic profiles of hydrophilic molecule (fluorescein) from lyotropic colloidal liquid crystals were studied, using Fickian’s law, which quantified the flux of drugs through a polymeric or lipid shells, as a function of time and physicochemical parameters of formulations, such as the drug distribution between the internal compartment and the external medium, the coefficients of diffusion and repartition, the thickness of the boundary layer, and the surface adsorption of drug. These parameters also modulated the release of the drugs from a bulk or colloidal matrix, in the aqueous compartment [3,25]. Furthermore, the Higuchi model was not applied to the full-range of the release profile [31].

Biopharmaceutical analysis showed that the fitting and predictions of results, obtained by applying Higuchi’s law, were somewhat different from those obtained by directly applying Fickian’s law, in the case of the colloidal carriers. These differences were observed by analyzing the mechanism of drug diffusion through the colloidal matrix, as well as the aqueous solution, and they were also affected by the interaction between the external surface of colloidal formulations and the internal compartment of colloidal carriers [32]. Our research group has previously demonstrated that a square root equation similar to Higuchi’s could be obtained by transforming the initial and the boundary conditions in solving Fick’s second law of diffusion [26]. Recent data also showed that this model could be widely used to investigate the release kinetics of dextran microspheres [33], poloxamer gels [34], and cylinder matrix systems [35].

Various scientists [3,36,37] have demonstrated that mathematical models could be classified in two different categories: (i) empirical, which were mainly used for fitting experimental data with a given power or exponential function, and (ii) phenomenological which considered physicochemical phenomena, such as mass diffusion transfer or processes of chemical reaction.

The drug release kinetic was also affected by the composition of carriers and technological parameters, such as excipients, materials, drug loading, geometry, size, and shape. Furthermore, for conventional formulations, the amount of drug (quantified after dissolution in the receptor medium), depended, both, on the payloads released in this compartment and on the bulk of drugs that were still entrapped inside the formulations [3]. In particular, this amount of drugs could be used to describe the release kinetic of formulations.

The extrapolation of theoretical criteria, reported herein for conventional formulations, allowed a description of the release kinetic profiles of the lyotropic colloidal liquid crystals, by considering the distribution of drug in two different compartments; particularly, for a small inner fraction (≤10%) and a sequestered fraction (≥40%). A small lag time could also be observed for the lyotropic colloidal liquid crystals, besides an equilibration time, which occurred between the supramolecular carrier and the receptor of the release apparatus.

### 3.1. Analysis of the Physicochemical and Technological Properties of Lyotropic Colloidal Liquid Crystals

The lyotropic colloidal liquid crystals had average sizes below 200 nm (Table 2). The payload did not affect the averages sizes of the lyotropic colloidal liquid crystals, which depended on the surfactants making the nanoformulations. Surfactants such as, polyethylene stearyl (Brij72), polyethylene oleyl (Brij 92) ethers, and various polyethylene or polypropylene block copolymers, made up the nanoformulations with the smaller average sizes than those containing polysaturated fatty acids, i.e., glyceryl monooleate (Table 2). The average sizes of formulations 1 and 5 were 162.4 and 148.3 nm, respectively; while formulations 3 and 4 were 187.3 and 183.4 nm, respectively (Table 2). The biggest sizes were obtained for formulation 2, which had an average size of 548.2 nm. In fact, it contained glyceryl mono-oleate derivatives with stearyl ether of polyoxyethilene (compared to formulations 3 and 4), instead of pluronic or symperonic co-polymers. This modification in the composition of lyotropic colloidal liquid crystals could affect their fluidity and allow a less stable rearrangement of the crystalline structure. Lyotropic colloidal liquid crystals, with an average size below 162 nm, were narrow size distributed, compared to the biggest ones (Table 2). The polydispersity index (PDI) of formulations 1 and 5 was below 0.17, it was increased over 0.22 for formulations 3 and 4 (Table 2). As expected, the biggest PDI was carried out for formulation 2. The increase of PDI over 0.2 could depend on the small crystal structures, which were formed in the colloidal suspensions as a consequence of the partial instability of the lyotropic colloidal liquid crystals having surfactants with polysaturated fatty acids in their backbone structure. This effect was specific for formulations 2–4 containing the Brij 72 and symperonic. These results were similar to those previously reported for ketoconazole-loaded lyotropic colloidal liquid crystals [38].

The loading of fluorescein inside the lyotropic colloidal liquid crystals did not affect their average sizes and narrow size distribution (Table 2).

Z-potential represents the measurement of the electro-kinetic potential of colloidal dispersions [39]. The Z-potential is the electric potential in the interfacial double layer (DL) at the location of the slipping plane, relative to a point in the bulk fluid, away from the interface, which represents the potential difference between the dispersion medium and the stationary layer of fluid attached to the dispersed particle. The Z-potential depends on the net electrical charge contained within the region, bounded by the slipping plane, and on the location of that plane. For this reason, Z-potential measures the magnitude of the charge and is not equal to the Stern potential or electric surface potential in the double layer [40], because it is quantified at different locations. The magnitude of the Z-potential also indicates the degree of electrostatic repulsion between charged nano- and micro- particles in a dispersion medium. This is the reason why colloids with a high negative or positive Z-potential are stable as a suspension. Z-potential is basically evaluated, experimentally, using the determined electrophoretic mobility or dynamic electrophoretic mobility, which depends on the dielectric constant of the solvent where the colloids are dispersed, and the surface composition. Electrophoresis is used for estimating the Z-potential of particulates; in particular, particles within the dispersion, which have a specific surface charge, migrate toward the electrode of opposite charge, with a velocity proportional to the magnitude of the Z-potential. Furthermore, particle concentration, shape and composition, as well as medium composition, can affect the Z-potential value and the theory applied for its measurements.

The Z-potential of aqueous or buffer dispersion is calculated, according to Smoluchowski’s theory which can be applied for colloidal nanoformulations, such as lyotropic liquid crystals, PEGylated liposomes, niosomes and PEGylated nanoparticles, having a thin double layer and a Debye length (1/κ) smaller than the particle radius. Additionally, polyethylene glycol or polyethylene/polypropylene co-polymers make the backbone of the structure of surfactants self-assembling into the lyotropic colloidal liquid crystals, which have properties and composition similar to that of polyethylene glycol. This is used to synthesize other different colloidal nanoparticles made up of organic or hybrid materials. These colloidal nanoparticles have a negative Z-potential, as has been previously reported [41,42,43,44,45,46], and the value of the negative charge depends on the medium in which the colloidal nanoparticles are diluted/dispersed, before the analysis, as well as other components of the pharmaceutical formulations [47,48,49,50,51,52,53,54].

The Z-potential values of lyotropic colloidal liquid crystals were below −29 mV, except in formulation 2 (Table 2). Formulations showing Z-potential values of +30 or −30 mV, or less lower, were stable and they did not make aggregates in aqueous suspensions [55,56,57]. Conversely, formulation 2, with the bigger sizes and wider size distribution than the other formulations (Table 2), had a Z-potential value of −15 mV and was unstable overtime. The instability of formulation 2 might have been due to the monoglyceryl oleate saturated fatty acid and the lack of polyoxyethylene and polyoxypropylene units in its structure. In fact, polyoxyethylene and polyoxypropylene units, making the stealth corona of the external layer of colloidal nanoparticles, increased the stability of lyotropic colloidal liquid crystals in biological fluids [58,59], as well as their long-circulation [60], and generated a steric hindrance hampering the aggregation [43,61] and macrophage uptake [62,63]. Polyoxyethylene and polyoxypropylene oxide block copolymer, coating the external layer of formulations 1, 3, 4, and 5, could also adsorb water on the surface of lyotropic colloidal liquid crystals, thus, increasing the ionic forces that affected the repulsion between polymeric chains and stabilized the nanoformulations. This effect of polyoxyethylene and polyoxypropylene block copolymers was further supported by electrophoretic mobility, which showed some surface modifications of the total surface energy when polyethylene and polypropylene oxide copolymers covered the surface of nanoparticles [46]. The loading of fluorescein did not affect the Z-potential values of lyotropic colloidal liquid crystals, thus, showing that the payload was entrapped in the aqueous compartment of nanoformulations and any payloads were adsorbed on the external surface.

A total of 0.03% (w/v) of fluorescein, which corresponded to 3.75 mg/mL, was used during the experiments. The lyotropic colloidal liquid crystals showed an entrapment efficiency percentage over 60% (Table 2). The following increasing order of entrapment efficiency percentage of fluorescein was obtained for the different formulations: Formulation 2 (87%, 3.27 mg/mL) > formulation 1 (85%, 3.19 mg/mL) > formulation 4 (75%, 2.81 mg/mL) > formulation 5 (64%, 2.4 mg/mL) > formulation 3 (60%, 2.25). These results were in agreement with the theoretical release kinetic experiments of lyotropic colloidal liquid crystals and endorsed differences of nanoformulations in terms of mathematical models.

### 3.2. Description and Analysis of the Obtained Release Kinetics

**Figure 1:**
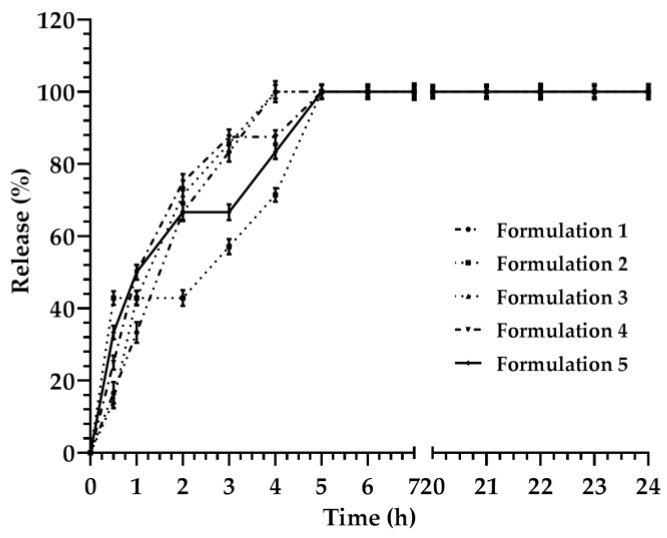
Release kinetic of fluorescein through lyotropic colloidal liquid crystal formulations. Lines and symbols, if not shown, are merged or filled in with those of other formulations. Data are the average of three different experiments ± standard deviation. Error bar if not shown is within the symbol.

The release kinetic of fluorescein from lyotropic colloidal liquid crystals was investigated based on their liquid crystal compositions (Table 1). Surfactants forming the lyotropic colloidal liquid crystals affected the release kinetic of various formulations (Figure 1), and lag times occurred for different formulations.

The release kinetic of formulations caused the steady state and the saturation of the medium, within the first six hours, in the range of incubation time from 0 to 24 h. Results were derived from the amount of fluorescein, which was released from lyotropic colloidal liquid crystals during the incubation time. Consequently, the mathematical model of the release kinetic profile was normalized to values obtained after 6 h of incubation, and the amount of fluorescein released at 6 h was considered as m_∞_, or equal to 100% of payloads released during this time (Figure 1).

A standard procedure for testing different cluster models was designed. The theoretical models already reported, e.g. linear, Higuchi, Noyes–Whitney, Siepman–Peppas, and Weibull, were selected, and the analyses were carried out with or without considering the lag time of the resulting data. The partial time or the full-range of the times were evaluated by fitting the theoretical models with the experimental data. In particular, a hierarchy of the fitting success was established by applying Akaike, Schwartz, and Imbimbo information criteria to experimental data. The statistical significance of differences between parent and degenerated models was tested using the *F*-test. The mechanistic component of these phenomenological models was selected as most reliable factor for the analysis fitted in the partial or full-range time of the experiments. The comparison of the best models resulting from the different formulations was carried out. The formulations, having similar data, were merged into a single model, although the active surface factors could lead to critical phenomena and significant changes of the structure of formulations.

**Figure 2:**
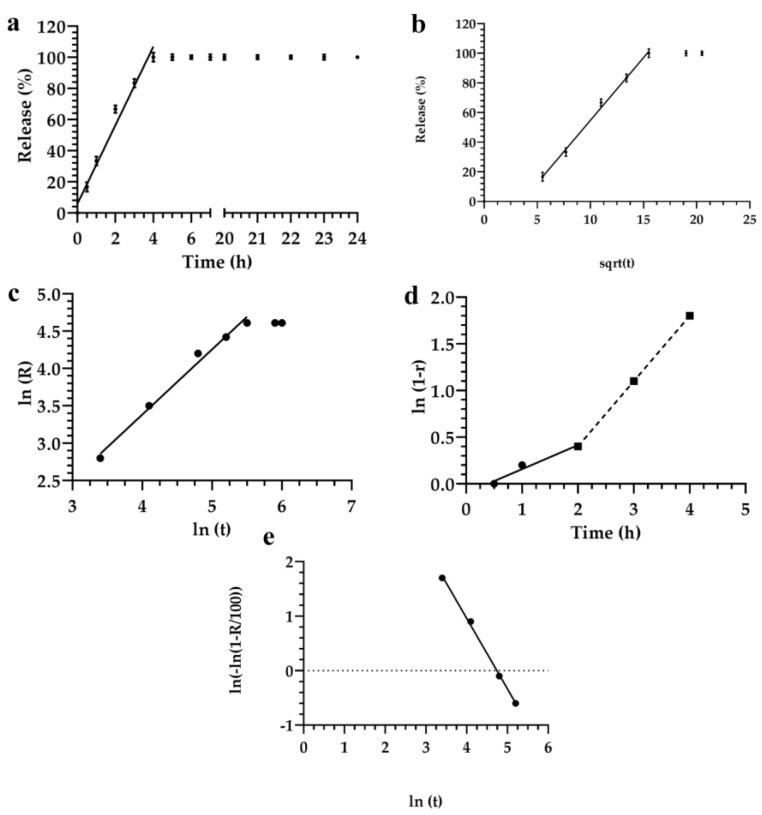
Release kinetic of fluorescein from the lyotropic colloidal liquid crystal formulation 1, according to the following mathematical models: (**a**) Linear regression; (**b**) Higuchi; (**c**) Siepman–Peppas; (**d**) Noyes-Whitney; (**e**) Weibul. Data represented by the average of three different measurements. Error bars if not shown are within the symbols.

Significant differences were observed for formulation 1 in the fitting performances deriving from the direct linear regression model and the Siepman–Peppas model, after their transformation and linearization. The release kinetic of formulation 1 showed that the sum of the squared errors remained lower (SS = 128) when the Siepman–Peppas model was applied, instead of the SS (140) obtained in the case of direct linear regression (Figure 2a,c, respectively).

The *r*^2^ value obtained by using the Siepman–Peppas model for data analysis was 0.988; while those obtained using the linear regression model was 0.975. Some values needed to be discarded because they were not included in the linear trend of the equation. Data analysis, obtained by applying the Fisher test and considering the linear model, as a degeneration of the power law, demonstrated that the increase of release kinetic showed a random effect. In fact, Siepman–Peppas law basically described the model release profiles of drugs from polymer, based on colloidal systems [64,65], which could be similar to our formulations.

By fitting the data with the Noyes–Whitney linear equation, showed that all mathematical models had different mechanisms of release in the first and in the last two hours. In fact, the release kinetic decreased after two hours of incubation (Figure 2d).

No further significant difference was observed when the Siepman–Peppas, and the Noyes–Whitney equations were applied for the analysis of data. The *r*^2^ values were similar for both models (Figure 2). The Higuchi model did not provide a better or worse model for the analysis of data, but it provided significant advantages by approximating a maximum number of experimental points.

For small values of αtβ, the Weibul model degenerated to the Higuchi or Siepman–Peppas models, $r(t)=1-e^{-\alpha t^{\beta }}\approx 1-(1-\alpha t^{\beta })\approx \alpha t^{\beta }$. In this case, it was possible to apply the *F*-test for evaluating the significance of the increase of fluorescein released from lyotropic colloidal liquid crystals. These data were fitted with those obtained using the Weibul model. However, these results did not completely satisfy this theoretical model, thus demonstrating that the Higuchi and the Siepman–Peppas models allowed a better extrapolation of data from experimental analysis than the Weibul model (Figure 2). The Higuchi model also provided some advantages for the fitting a large number of experimental data, and increased the prediction power and the statistical significance of the analysis.

**Figure 3:**
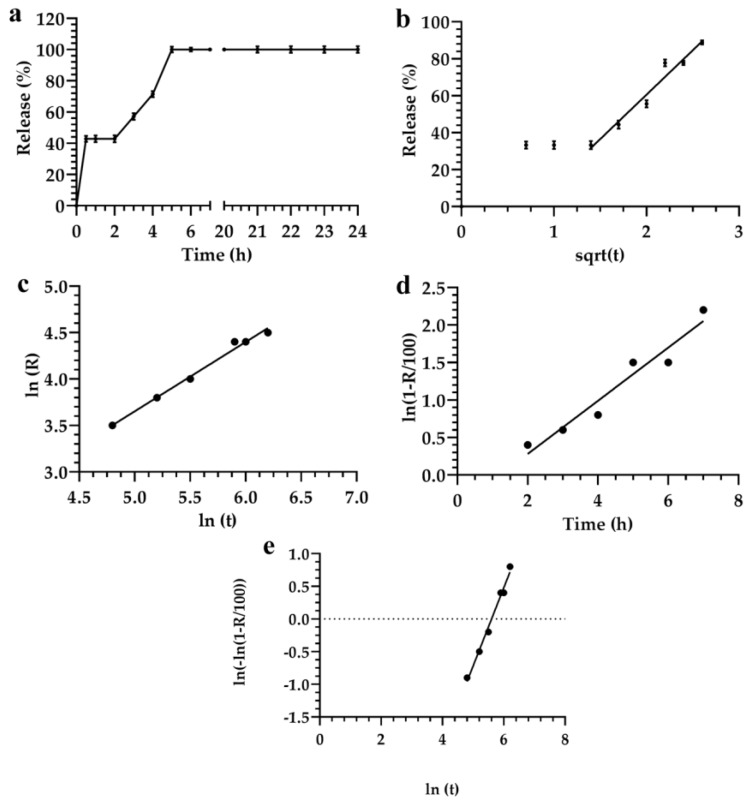
Evaluation of the release profile of fluorescein from the monoglyceride colloidal liquid crystal formulation 2, using the following mathematical models: (**a**) Linear regression; (**b**) Higuchi; (**c**) Siepman–Peppas; (**d**) Noyes–Whitney; and (**e**) Weibull. Data represented the average of three different measurements. Error bars, if not shown, are within the symbols.

The mathematical analysis of formulation 2 showed that the release profile of fluorescein using Higuchi, Siepman–Peppas, and Weibull models could be suitably evaluated by starting from 2 h and ending the analysis after 7 h (Figure 3).

Results obtained using the statistical criteria did not provide the best model for describing the release kinetic of data reported herein (Figure 3).

Significant differences of parameters, that were analyzed using the selected mathematical models, could be affected by the surfactant compositions of the nanocarriers [31].

The Akaike and Schwarz criteria showed that the Weibull equation represented the best equation to analyze data in the range of time 2–6 h. This hypothesis agreed with data previously reported [10,66]. Conversely, the Imbimbo criterion showed that the Higuchi model provided detailed information about the release kinetic of fluorescein. Bhaskar et al. also obtained similar results for nitrendipine released from solid lipid nanoparticles and nanostructured lipid carriers [67].

No significant difference was obtained by using the *F*-test analysis of the models reported previously. In particular, the *F*-test showed that data analyzed using the Siepman–Peppas model was more significant than those using the other models (Figure 3c). In fact, the *r^2^* value of this model was 0.991, and all data fitted with the linear trend of the applied equation. The correlation coefficients *r*^2^ obtained by using Weibull, Higuchi and Siepman–Peppas models had values of 0.956, 0.993, and 0.991, respectively. The Imbimbo comparison of Higuchi and Weibull models of the release kinetic profile of formulation 2 did not show any significant difference (Table 3). This result showed that the Higuchi model could be preferentially used for the analysis of data because it was easy to use and provided a better phenomenological analysis of data than the other models. Conversely, the Higuchi model can only be applied for the first part of the experimental set of data.

**Figure 4:**
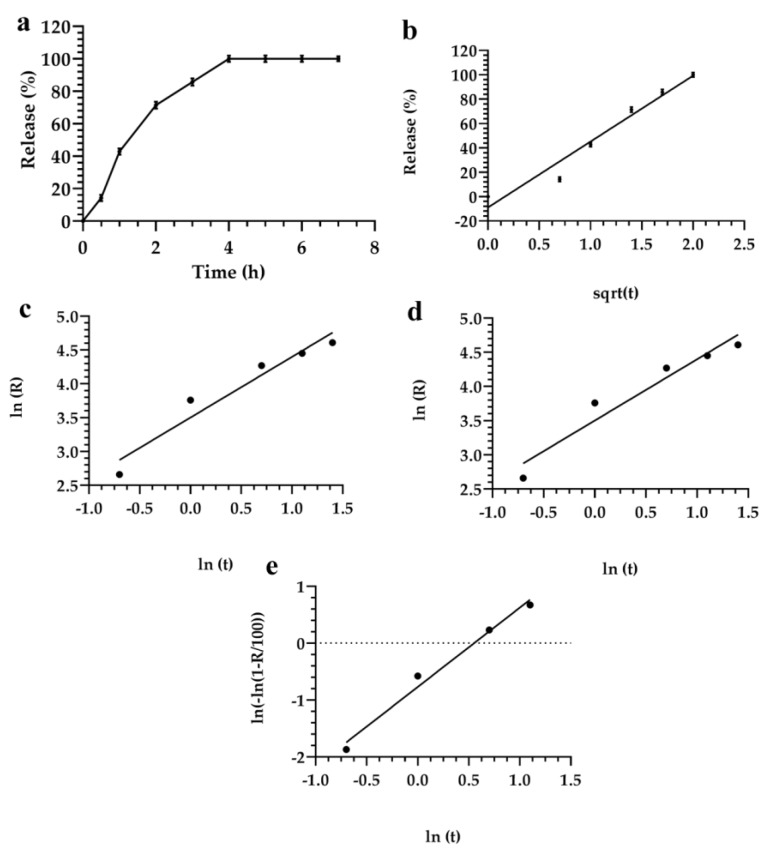
Evaluation of the release profile of fluorescein from the lyotropic colloidal liquid crystal formulation 3, using the following mathematical models: (**a**) Linear regression; (**b**) Higuchi; (**c**) Siepman–Peppas; (**d**) Noyes–Whitney; and (**e**) Weibull. Data represented the average of three different measurements. Error bars, if not shown, are within the symbols.

The release kinetic profile of formulation 3 showed that the fluorescein was rapidly released from lyotropic colloidal liquid crystals after 4 h, while a plateau occurred from 4 h up to 24 h (Figure 1). A biphasic profile of the release kinetic was obtained for formulation 3, and it was represented by two different models, i.e., the first part (0–4 h), which was linear, and the second part (4–24 h), which was saturated. The sums of the standard errors and the correlation coefficients demonstrated that the mathematical correlation was suitable for the analysis of data, while no significant difference occurred in the linear part of the release kinetic of fluorescein for the Siepman–Peppas and Higuchi models (Figure 4). The *F*-test analysis did not show any statistically significant increase of the curve when the mathematical model was changed from the Siepman–Peppas to the Weibul (Figure 4c–e). The direct linear fitting dependence demonstrated a poor correlation between the resulting data (Figure 4a).

All mathematical models allowed the fitting of data in the first 6 h, except the Siepman–Peppas and the Weibull models that could be used to fit the results in the full range of analysis. The Noyes–Whitney model was applied to analyze formulation 3 and got the best fitting of results (Figure 4d).

**Figure 5:**
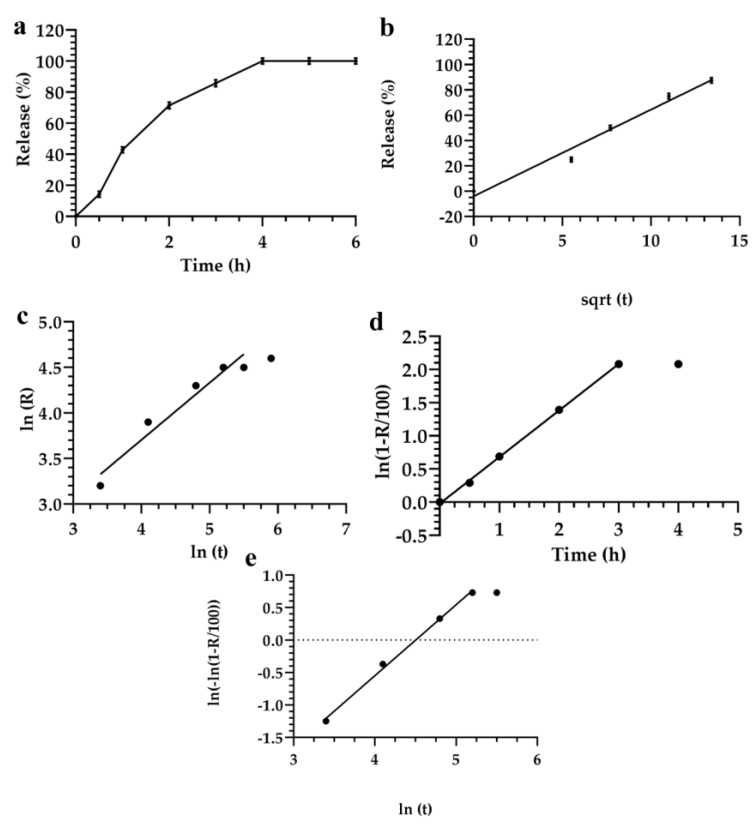
Evaluation of the release profile of fluorescein from lyotropic colloidal liquid crystal formulation 4, using the following mathematical models: (**a**) Linear regression; (**b**) Higuchi; (**c**) Siepman–Peppas; (**d**) Noyes–Whitney; and (**e**) Weibull. Data represents the average of three different measurements ± standard deviation. Error bars, if not shown, are within the symbols.

The mathematical analysis of the release profile of formulation 4 showed that the Noyes–Whitney model did not allow predicting the release of fluorescein from lyotropic colloidal liquid crystals in physiological solution and aqueous media (Figure 5). For this reason, the release kinetic of formulation 4 was evaluated using the Higuchi model. Results obtained by applying the Higuchi model (Figure 5b) showed that this mathematical model could be used to analyze the release of fluorescein in the first 3 h (Figure 1). By extending the release analysis up to 5 h, the Siepman–Peppas and Weibul empirical models were applied (Figure 5c,e). Both models increased the statistical significance of the analysis for the release kinetic of formulation 4, thus demonstrating that Siepman–Peppas semi-empirical and the Weibull empirical models (Figure 5b,c) obtained similar results. These results enforced the hypothesis that the Siepman–Peppas model could be applied to predict the release kinetic of fluorescein from formulation 4 (Figure 5c).

**Figure 6:**
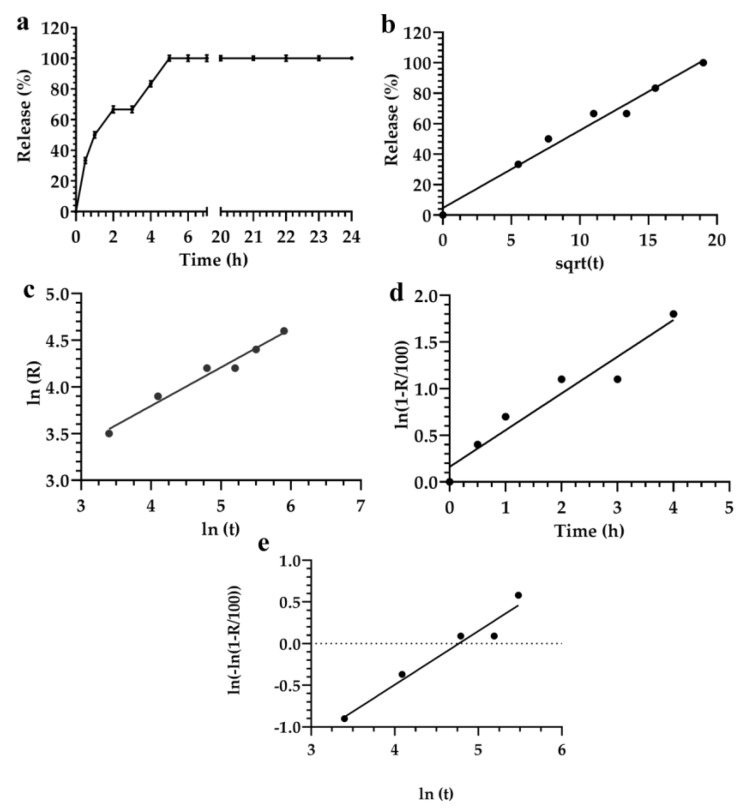
Evaluation of the release profile of fluorescein by lyotropic colloidal liquid crystal formulation 5, using the following mathematical models: (**a**) Linear regression; (**b**) Higuchi; (**c**) Siepman–Peppas; (**d**) Noyes–Whitney; and (**e**) Weibull. Data represents the average of three different measurements ± standard deviation. Error bars, if not shown, are within the symbols.

Different results were obtained for formulation 5. In this case, the Higuchi model showed that a specific fitting occurred when the analysis was carried out up to 20 h of incubation (Figure 6). Theoretical and experimental analyses were strictly correlated when applying the Higuchi model. The Higuchi model also correlated the theoretical and experimental analyses. No significant differences of the release kinetic of various formulations were obtained by analyzing data with linear regression, the Siepman–Peppas, Weibull, Higuchi, and the Noyes–Whitney models (Figure 6).

Results showed that by applying the square root model data, theoretical and experimental data was fitted for five different formulations; and this model increased the statistical significance of experimental data, compared to the linear regression.

The linear regression of formulations (1–5) demonstrated that the data had the same trend and similar *r*^2^ values (Supplementary Figure S1), while the *t*-test significance of results was calculated by comparing the equations reported earlier (Supplementary Information) [68].

The linear regression of lyotropic colloidal liquid crystals was also parallel for formulations 1, 3, 4, and 5, and demonstrated that the minimum and maximum slopes were the same for a *p* value of 0.77 (Supplementary Figure S1). This value was considered to be acceptable for the mathematical models used during the analysis. Conversely, the formulation 2 had a *p* value ≥ 0.77; this result provided a delay time of fluorescein release from lyotropic colloidal liquid crystals in the first part of the curve, and a rapid release of payload in the second part of curve (Figure 1).

The parallelism of the regression line calculated for the square root of formulations 1–5 demonstrated that this model was robust for describing the release kinetic of fluorescein from lyotropic colloidal liquid crystals and its diffusion through the various formulations, which depended both on the structure and physicochemical properties of the surfactants. The analysis of the release kinetic of fluorescein from the lyotropic colloidal liquid crystals, depending on the initial and boundary conditions, provided some advantages for setting-up the parameters of mathematical models and designing the suitable mathematical models for the analysis.

The lyotropic liquid crystals also had a first order release kinetic with a significant burst effect when hydrophilic payloads were included in their supramolecular structure [69,70,71]. This effect might depend on the shape of the formulations, the loading of payloads in their aqueous channels, as well as its adsorption on their external surface. The burst release effect of hydrophilic drugs from the lyotropic liquid crystals could be also explained at the theoretical level, using the Higuchi square root model [72]. In fact, hydrophilic drugs with different molecular weights, such as fluorescein, could be loaded into a liquid crystalline aqueous compartment and provided a cumulative drug release through the surfactant membrane, by a linear relationship between drugs and the square root of time, which has been also considered to be the Higuchi diffusion-controlled release kinetic [9]. Previous data demonstrated that the release of diclofenac salts from lyotropic liquid crystalline nanocarriers [73], as well as the release of charged molecules from lipid cubic phases, in a quasi-equilibrium process [74], and that of doxorubicin from pH-sensitive lipid cubic phase matrices [75], were described using the Higuchi law.

An in-depth phenomenological approach demonstrated that the release of hydrophilic drugs from liquid crystalline mesophases could depend on the diffusion, the accumulation and the water/lipid partition coefficient at the interface of colloidal nanoparticles [76]. Similar results were obtained for the release kinetic of Aloe vera loaded cubogels used in the treatment of deep second-degree burns [77].

The release kinetic of different lyotropic colloidal liquid crystals could be solved using Higuchi’s law, although this mathematical model needed some specific conditions to be applied. Additionally, the diffusion of a solvent through nanocarriers (made up of surfactants) was different from that of the solid drug dosage form, and lipid or polymeric nanoparticles. In fact, the release kinetic of payloads from lyotropic colloidal liquid crystals containing surfactants depended on the square root of time [78,79,80]. Lyotrophic phases of the colloidal liquid crystals accumulated the payloads in the aqueous compartment and provided a zero order release kinetic specific to a reservoir system. This model of diffusion depended on the drug concentration as well as the square root of time. Conversely, the release kinetic of hydrophilic payloads from the solid dosage form and from polymeric nanoparticles, showed a release kinetic model similar to that obtained by applying the Higuchi model. This property depended on the permeation of the solvent in the internal matrix and the resulting dissolution of the powders of polymers; however, this was not the case for lyotropic colloidal liquid crystals.

## 4. Conclusions

Results demonstrated that the analysis of the release profile of lyotropic colloidal liquid crystal formulations were characterized by a continuous release of fluorescein up to 6 h, with the exception of formulation 3, which showed a rapid release during the first hour followed by a gradual and continuous release up to 6 h. The trend of the release kinetic did not show a linear property, since the saturation phase occurred for all of the formulations analyzed. This was due to a complete release of fluorescein from the lyotropic colloidal liquid crystals.

The theoretical data calculated by applying the mathematical models can be used to predict the experimental release kinetic profile of fluorescein from the lyotropic colloidal liquid crystals. These mathematical models can also be applied for the validation process. This model was not affected by the chemical compositions or structure of nanocarriers. The general information obtained by applying the square root model could be further improved by applying the Weibull and Siepman–Peppas models. In fact, these two models allowed for obtaining precise information concerning the release profile of fluorescein from lyotropic colloidal liquid crystals. Moreover, both of these models represented a valid alternative to the square root model, and can be easily applied and directly calculated using the diffusion equation. The composition of lyotropic colloidal liquid crystals did not affect the release kinetic profile of the fluorescein, and the resulting linear regression of the release profile, as a function of the square root of time, was the same for the four of five formulations analyzed.

## Figures and Tables

**Table 1:** Chemical compositions of lyotropic colloidal liquid crystals.

Formulations	Monomuls	Brij 72	Brij 92	Pluronic 10500	Pluronic F68	Symperonic
1	—	—	90 mg	—	50 mg	—
2	90 mg	50 mg	—	—	—	—
3	90 mg	—	—	—	—	50 mg
4	90 mg	—	—	50 mg	—	—
5	—	—	90 mg	—	—	50 mg

**Table 2:** Physicochemical characterization of lyotropic colloidal liquid crystals.

Formulations	Size (nm)	PDI ^2^	Z.P. (mV) ^3^	E.M. (μm × cm/Vs) ^4^	E.E (%) ^5^
1	162.4 ± 1.3	0.16 ± 0.05	−29.3 ± 1.5	−2.1 ± 0.2	−
1 + FL ^1^	163.5 ± 2.1	0.18 ± 0.03	−30.3 ± 0.5	−2.1 ± 0.3	85.2 ± 3.1
2	548.2 ± 0.9	0.35 ± 0.09	−15.4 ± 0.7	−1.79 ± 0.1	−
2 + FL ^1^	550.4 ± 1.5	0.37 ± 0.06	−16.3 ± 0.4	−1.82 ± 0.1	87.1 ± 2.9
3	187.3 ± 1.5	0.24 ± 0.07	−30.2 ± 1.4	−2.39 ± 0.18	−
3 + FL ^1^	188.8 ± 1.7	0.26 ± 0.02	−32.2 ± 0.9	−2.42 ± 0.15	61.9 ± 4.1
4	183.4 ± 6.1	0.25 ± 0.09	−33.1 ± 1.5	−2.12 ± 0.21	−
4 + FL ^1^	185.2 ± 2.5	0.27 ± 0.1	−34.9 ± 1.3	−2.15 ± 0.18	75.1 ± 5.1
5	148.3 ± 1.6	0.14 ± 0.03	−30.5 ± 1.4	−2.3 ± 0.06	−
5 + FL ^1^	150.1 ± 1.6	0.16 ± 0.07	−31.9 ± 1.7	−2.5 ± 0.1	65.9 ± 5.7

^1^ FL = fluorescein; ^2^ PDI = polydispersity index; ^3^ Z.P. = Z-potential; ^4^ E.M. = electrophoretic mobility; ^5^ E.E. (%) = entrapment efficiency (%).

**Table 3:** Comparison of different mathematical models for evaluating the release kinetic of fluorescein from lyotropic liquid crystal.

Mathematical Model	Akaike	Schwarz	Imbimbo	*F*-test
Higuchi	29.7	31.3	0.057	0.242
Weibul	25.8	25.4	0.059	—

## Supplementary Materials

The following are available online at https://www.mdpi.com/1996-1944/12/5/693/s1, Figure S1: *t*-test applied to compare the linear regression for square root equation for formulations 1, 3, 4, and 5; Equations S1–S10.

## Abbreviations

(Brij® 92 or Brij O2)	Polyethylene glycol oleyl ether
(Brij® 72)	Polyoxyethylene stearyl ether
(Symperonic® PE/F127)	Polyoxyethylene-polyoxypropylene block copolymer 127
(Pluronic® PE 6800)	Polyethylene glycol-polypropylene glycol-polyethylene glycol block copolymer 6800
(Pluronic® PE 10500)	Polyethylene glycol block copolymer 10500
(Monomuls® 90-O18)	Glyceryl mono-oleate
(NaCl 0.9 % w/v)	Isotonic sterile saline solution
FL	(Fluorescein)
PDI	(Polydispersity index)
Z.P.	(Z-potential)
E.M.	(Electrophoretic mobility)
E.E. (%)	(Entrapment efficiency (%)).
