# Supplementary material for: Mathematical Models as Tools to Predict the Release Kinetic of Fluorescein from Lyotropic Colloidal Liquid Crystals
Source: Materials (Basel). 2019 Feb 26;12(5):693. doi: 10.3390/ma12050693 (PMC6427212; doi:10.3390/ma12050693)
Supplement: Supplementary file 1 [file materials-12-00693-s001.pdf]

*materials*

# Mathematical Models as Tools to Predict the Release Kinetic of Fluorescein from Lyotropic Colloidal Liquid Crystals

**Donatella Paolino [1], Andra Tudose [1,2], Christian Celia [2,3,*], Luisa Di Marzio [3], Felisa Cilurzo [3] and Constantin Mircioiu [2,*]**

1 Department of Experimental and Clinical Medicine, University of Catanzaro "Magna Graecia", Viale "S. Venuta" s.n.c., 88100 Catanzaro, Italy; paolino@unicz.it
2 Department of Applied Mathematics and Biostatistics, Faculty of Pharmacy, University of Medicine and Pharmacy “Carol Davila” Bucharest, 6 Traian Vuia, 020956, Bucharest, Romania; andratds@yahoo.com (A.T.)
3 Department of Pharmacy, University of Chieti - Pescara "G. d'Annunzio", via dei Vestini 31, 66100 Chieti, Italy; luisa.dimarzio@unich.it (L.Di M.); felisa.cilurzo@unich.it (F.C.)
* Correspondence: c.celia@unich.it (C.C.), constantin.mircioiu@yahoo.com (C.M.); Tel.: +39 0871-355-4711 (C.C.), +40 723-175-022 (C.M.)

**Keywords:** lyotropic colloidal liquid crystals; release profile; mathematical models; drug delivery systems; release kinetic; square root laws

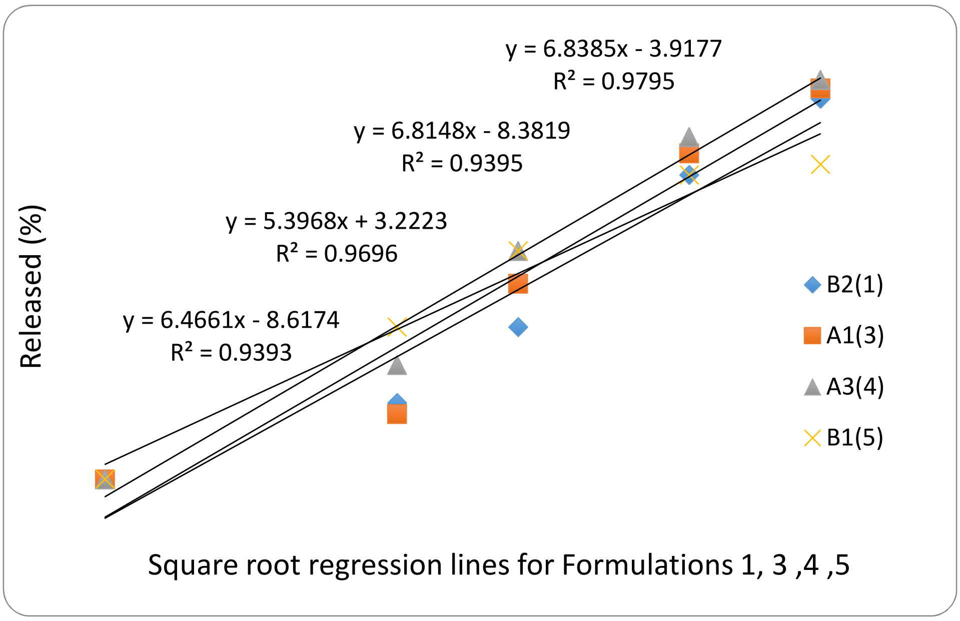


**Supplementary Figure 1.** *t*-test applied to compare the linear regression for square root equation for Formulations 1, 3, 4, and 5. The slope of Formulation 2 is below 0.7 and this formulation could be excluded from the *t*-test analysis.

**Slopes of the Different Formulations**

Equation S1:

$$T_{n_1+n_2-3} = \frac{(a_1 - a_2) - b(\overline{x_1} - \overline{x_2})}{S\sqrt{\frac{1}{n_1} + \frac{1}{n_2} + \frac{(\overline{x_1} - \overline{x_2})^2}{\sum(x_{i1} - \overline{x_1})^2 + \sum(x_{i2} - \overline{x_2})^2}}}$$

The extreme values of slopes were 5.39 and 6.83, respectively; while it was 0.77 for only Formulation 2.

$$H_0: \quad \alpha_1 + \beta_1 x \equiv \alpha_2 + \beta_2 x$$

A probability greater than 0.95 showed that $H_0$ hypothesis was in the linear range, was similar for different formulations, and could not be rejected, except for Formulation 2, which had a *p* value of 0.77.

**Solution of Diffusion Equations for the Release Kinetic from a Reservoir System in a Semi-Infinite Medium**

The release kinetic of a reservoir system in a semi-infinite medium depends on the heat transfer, the impulse of the fluid movement, and the probability of quantum mechanics of single molecules. In this attempt, the diffusion equation of this system could be calculated using the following equation:

$$\square_a u(x_1, x_2, \ldots x_n; t) = 0 , \tag{S2}$$

where, $\square_a$ is the operator

$$\square_a = a\Delta - \frac{\partial}{\partial t} , \tag{S3}$$

A fundamental result of equation S3 was the theoretical solution obtained when one unit of the mass was released in one unit of time, similar to that of intravenous bolus administration:

$$a\frac{\partial^2 u(x;t)}{\partial x^2} - \frac{\partial u(x;t)}{\partial t} = \delta(x) \times \delta(t) = \delta(x;t) , \tag{S4}$$

By applying the Fourier transformation to the parameter *u (x,t)* for *x*, and then the Laplace transformation as function of *t*, it was possible to obtain the following equation:

$$-\alpha^2 a L(F(u)) - pL(F(u)) = 1(\alpha) \times 1(p) = 1 , \tag{S5}$$

Based on the equation S5, the double transformation of *u (x,t)* could be reported using the following equation:

$$L(F(u)) = -\frac{a}{p + a\alpha^2}, \quad \text{(S6)}$$

By applying the inverse of the Laplace function, Equation S6 was transformed into the following equation:

$$F[u(x;t)] = -aH(t)e^{-a\alpha^2 t}, \quad \text{(S7)}$$

where, $H(t)$ is the Heaviside function.

The different solutions of Fick's second law depends on the initial and boundary conditions. In fact, it is difficult to quantify using an experimental model and the concentrations of drugs in liquid medium at the same time. Conversely, it is possible to theoretically estimate their distribution in semisolid media and transfer the course, by using an experimental Franz diffusion cell model.

When drug concentration is slightly affected by time, it is possible to calculate the amount of drug at the steady state. However, mathematical model of solid pharmaceutical formulations generally generates an independent convection flow, in order to homogenize the concentration of drugs in the release medium and to approximately obtain the sink conditions.

By further applying the inverse of Fourier transformed functions, Equation S7 was further transformed as follows:

$$E(x;t) = -a\frac{H(t)\sqrt{a}}{2\sqrt{\pi t}}e^{-\frac{x^2}{4at}}, \quad \text{(S8)}$$

Based on Equation S8, the square root of time could be transformed in a general equation. For the lyotropic colloidal liquid crystals, showing a rapid release of hydrophilic payloads, the release kinetic of drugs depended on their diffusion across the membrane of formulations. The internal compartment (internal interface) of the membrane provided a rapid release of hydrophilic payloads in the first hours of the kinetic process. Conversely, the latest hours of the release kinetic depended on the drug concentration and the kinetic process was modulated by the diffusion of drug in the released medium.

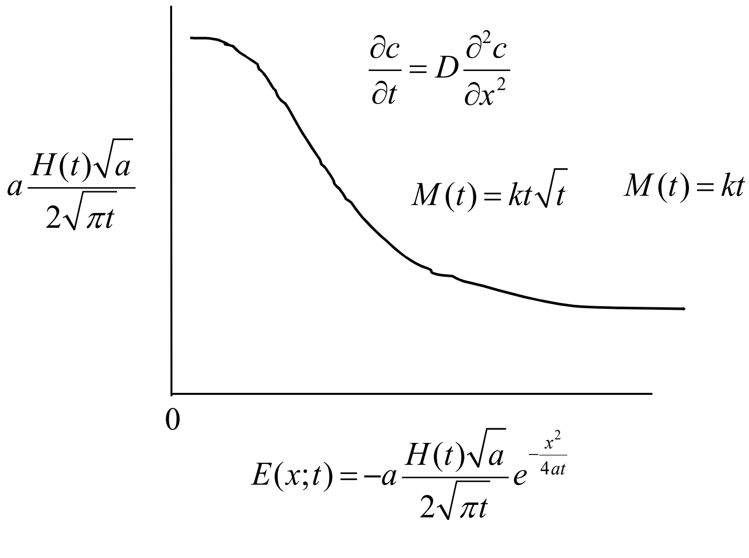


**Supplementary Figure 2.** Equation describing the release kinetic of fluorescein through the lyotropic colloidal liquid crystals as a function of drug concentration and kinetic process.

In the general model reported here, the release of drug through the interface of the internal compartment of the membrane, depended on bi-dimensional diffusion models, as follows:

$$E(x,y;t) = -\frac{\theta(t)}{4\pi t} e^{-\frac{r^2}{4at}}, \quad r = \sqrt{x^2 + y^2} \quad \text{(S9)}$$
,

and three-dimensional diffusion model, as reported in the following equation:

$$E(x,y,z;t) = -\frac{\theta(t)}{8\pi t\sqrt{\pi a t}} e^{-\frac{R^2}{4at}}, \quad R = \sqrt{x^2 + y^2 + z^2} \quad \text{(S10)}$$
,

In both models, *a* symbol was reported as *D*.

The amount of drug released from the lyotropic liquid crystals was proportional to $\sqrt{t}$ ,or *t*, or $t\sqrt{t}$ .

These data can be interpreted as mono-dimensional, bi-dimensional, or three-dimensional diffusion models.
